# Examining the Relationship between Youth-Targeted Food Marketing Expenditures and the Demographics of Social Media Followers

**DOI:** 10.3390/ijerph17051631

**Published:** 2020-03-03

**Authors:** Pasquale E. Rummo, Omni Cassidy, Ingrid Wells, Jaime A. Coffino, Marie A. Bragg

**Affiliations:** 1 Department of Population Health, New York University School of Medicine, New York, NY 10016, USA; pasquale.rummo@nyulangone.org (P.E.R.); Omni.Cassidy@nyulangone.org (O.C.); iw432@nyu.edu (I.W.); 2 Department of Psychology, University at Albany, State University of New York, Albany, NY 12222, USA; jcoffino@albany.edu; 3 Department of Psychiatry and Human Behavior, Warren Alpert Medical School, Brown University, Providence, RI 02912, USA; 4 Department of Nutrition, New York University School of Global Public Health, New York, NY 10012, USA

**Keywords:** social media, food marketing, targeted advertising, adolescents, sugar-sweetened beverages, fast food

## Abstract

*Background*: To determine how many adolescents follow food/beverage brands on Instagram and Twitter, and examine associations between brands’ youth-targeted marketing practices and percentages of adolescent followers. *Methods*: We purchased data from Demographics Pro to characterize the demographics of Twitter and Instagram users who followed 27 of the most highly advertised fast food, snack, and drink brands in 2019. We used one-sample *t*-tests to compare percentages of adolescent followers of the selected brands’ accounts versus all social media accounts, independent samples *t*-tests to compare followers of sugary versus low-calorie drink brands, and linear regression to examine associations between youth-targeted marketing practices and the percentages of adolescent followers. *Results*: An estimated 6.2 million adolescents followed the selected brands. A higher percentage of adolescents followed the selected brands’ accounts (9.2%) compared to any account on Twitter (1.2%) (*p* < 0.001), but not Instagram. A higher percentage of adolescents followed sugary (7.9%) versus low-calorie drink brands (4.3%) on Instagram (*p* = 0.02), but we observed the opposite pattern for adults on Twitter and Instagram. Television advertising expenditures were positively associated with percentages of adolescent followers of the selected brands on Twitter (*p* = 0.03), but not Instagram. *Conclusions*: Food and sugary drink brands maintain millions of adolescent followers on social media.

## 1. Introduction

Poor diet places youth at increased risk of excessive weight gain and chronic health conditions (e.g., type 2 diabetes, heart disease) during adulthood [1]. Food marketing is a major contributor to poor diet among children and adolescents [2,3], and companies spend approximately US$2 billion on youth-targeted food and beverage marketing across multiple forms of media (e.g., television [TV], the Internet) [4]. Additionally, nearly half of those expenditures (US$77 million) is spent on food advertisements that are placed on websites and digital platforms [4]. This investment in digital marketing has grown in tandem with consumers’ increasing engagement with online forms of media [5,6,7]. In 2018, for example, approximately 95% of adolescents (13–17 years) owned or had access to a smartphone, and almost 90% of adolescents reported using the Internet multiple times per day [8]. Seventy–one percent of adolescents in the United States report using the Internet to access Instagram, for example, [8], which is concerning given nearly 50% of adolescents report being online “almost constantly” [8]. These data underscore a shifting digital landscape where social media use is quickly outpacing or even displacing other types of media use [9,10,11].

Food and beverage companies have become sophisticated in their ability to target youth through social media [6]. Although social media ads use methods that are similar to TV advertising practices (e.g., celebrity endorsements [12]), many social media ads capitalize on techniques that are unique to digital platforms. This new digital era provides companies with the opportunity to directly interact with, track, and target youth through novel methods (e.g., creating free social media accounts where they can regularly post brand-specific content) [13,14]. Instagram, for instance, allows food and beverage companies to post photos accompanied by captions. When a social media user chooses to “follow” a food or beverage company’s social media account on sites like Instagram or Twitter, they can see brand-related photos, messages, and captions alongside posts by friends and family in their social network. Youth can also engage more directly with brands by “liking,” commenting, or sharing the company’s posts with others, and these interactive features are unique to social media marketing [15,16]. More data are needed to understand the ways youth engage with food and beverage companies on social media platforms.

The effects of exposure to food and beverage marketing on attitudes and behaviors are well-established [17]. Food and beverage ads lead to brand recognition and preferences for advertised foods [18], and children have positive attitudes towards advertised foods [19]. These effects are particularly problematic given that the majority of the food and beverages marketed to youth are predominately foods high in fat, sugar, and salt [20,21]. A number of lab-based studies have also shown that children who see food ads consume more calories than children who see non-food ads, and this increased caloric intake even occurs for foods that were not shown in the ads [19].

Although a growing body of research demonstrates that youth are bombarded with food and beverage ads [22,23,24,25], less is known about youth-targeted marketing on social media. To our knowledge, no study has examined youth’s engagement with food and beverage companies on social media platforms. To address these gaps in the literature, we analyzed Instagram and Twitter data to answer the following research questions:

1. What is the percentage and number of adolescents ages 13–17 years who follow food and beverage brands on Instagram and Twitter?
2. Do food/beverage brand accounts have a higher percentage of adolescent followers compared to all social media accounts on Instagram and Twitter? Do these associations differ for sugary drink brands relative to low-calorie drink brands?
3. Are youth-targeted food marketing practices associated with the percentage of adolescents who follow food and beverage brands on Instagram and Twitter?

## 2. Materials and Methods

### 2.1. Data

In 2016, we identified 30 fast food, snack, and beverage brands with the highest advertising expenditures [23,24,26]. We chose those three categories because they represent the products that are targeted most heavily to adolescents [20]. We then contacted Demographics Pro to inquire about the availability of data on the demographic characteristics of social media users who followed those 30 brands. Demographics Pro is a data analytics firm that uses proprietary algorithms to estimate or infer the demographic characteristics of target audiences based on social media activity (e.g., the frequency of posts). The firm filters and amplifies data signals from users’ networks (e.g., the strength of ties between individuals), consumption (e.g., accounts followed), and language (e.g., words used in posts). Their data and methods have been published in other types of public health research [27,28,29]. Demographics Pro has used their methodology to profile more than 300 million social media users. They require confidence of 95% or above to make an estimate for each demographic characteristic on a given social media profile. Their analytic predictions rely on the low covariance of multiple amplified signals. The firm evaluates their methods by iteratively testing their approach among established samples of social media users with verified demographics. Those established sample sizes include between 10,000 and 200,000 social media users with verified demographic information. This validation process enables more precise calibration of their methods. Their methods perform equally well when evaluating the demographic characteristics of Twitter users and Instagram users. At the time of the purchase, Demographics Pro had data on Instagram and Twitter users for 27 of the 30 brands, including 24 brands on Instagram and 19 brands on Twitter Table 1. We downloaded and analyzed the data in January 2019.

To identify which brands in our sample target child and adolescent consumers, we reviewed the University of Connecticut Rudd Center’s report on targeted food advertising from 2019 [22]. In addition to total TV spending, the report provides a Child:Adult targeted ratio and an Adolescent:Adult targeted ratio. We recorded the ratios for the brands in our sample, though data were not available for 9 of the brands we selected Table 2. The targeted ratios are calculated by dividing the ratio of TV ad exposure for children/adolescents versus adults by the ratio of TV viewing times, which accounts for racial differences in the time spent viewing ads. For example, an Adolescent:Adult targeted ratio of 2.00 indicates that adolescents viewed 100% more TV ads for a specific brand compared to adults, accounting for differences in TV viewing. These data are not available for social media, so we used spending on TV ads and disproportionate exposure to TV advertising as proxies for spending on social media ads and disproportionate exposure to social media advertising, respectively.

### 2.2. Outcomes

Our primary outcome in all analyses was the average percent of the followers of all food and beverage brands in our sample per age group per social media platform. We also separately calculated and compared the average percent of followers of fast food brands, all beverage brands, sugary drink brands, and low-calorie drink brands per age group per social media platform Table 2. We classified zero-calorie beverages and/or beverages with artificial sweeteners as low-calorie drink brands.

### 2.3. Statistical Analysis

For all of the food and beverage brands in our sample, we reported the mean (SD) of the percentage of their followers by age subgroup for both Instagram and Twitter. For each brand, we also reported the total number of followers on each social media platform as well as social media behaviors of followers, including posting frequency, the length of time since account creation, and geo-enabled settings (i.e., a setting that social media users can enable that allows the social media site to collect, store, and utilize the user’s precise location). We used one sample *t*-tests to examine whether the mean percentage of followers of brands in our sample was significantly different from the mean percentage of users who followed any account on Instagram or Twitter by age group. We used an independent samples *t*-test to determine whether the mean percentage of followers of sugary drink brands was significantly different from the mean percentage of followers of low-calorie drink brands. Finally, we used a linear regression model to examine the association between TV advertising spending and child/adolescent targeted ratios with the percentage of followers per age subgroup, by social media platform. We used Stata version 15 for all analyses [30].

## 3. Results

As of January 2019, there were an estimated 73.1 million social media users of any age who followed this sample of food and beverage brands on Instagram and/or Twitter. These brands had more followers on Instagram (*n* = 55.9 million) than Twitter (*n* = 17.2 million), though these figures may include users who followed multiple food/beverage brand accounts on both social media platforms Table 1. The majority of social media users who followed brands in our sample were located in the United States (55.6% for Instagram; 77.7% for Twitter). More than 29 million females of any age followed the brands in our sample on Instagram, and nearly 9 million females followed the brands on Twitter Table 2. Red Bull, Monster Energy, and Mountain Dew had the lowest percentage of female followers on Instagram (<23% each), whereas disproportionately high percentages of females followed Starbucks (76.9%), Diet Coke (70.8%), and Smart Water (65.9%) on Instagram. We found similar patterns among females and males who followed brands in our sample on Twitter. And an estimated 28.1 million Instagram users and 12.3 million Twitter users who followed the brands in our sample had their profile for more than 2 years. And many of these were active on social media: 22.9 million Instagram users and 6.6 million Twitter users who followed brands in our sample posted 1–7 times per week. Geo-network enabled settings were activated among an estimated 17.7 million Instagram users and 6.8 million Twitter users who followed brands in our sample.

### 3.1. Adolescent Followers of Food/Beverage Brand Accounts versus Any Social Media Account

An estimated 6.2 million adolescents followed the selected food and beverage brands on Instagram and/or Twitter Table 2. Approximately 3.8 million of those adolescents followed the 24 brands for which data were available on Instagram, whereas 2.4 million adolescents followed the 19 brands for which data were available on Twitter. 

On Twitter, the percentage of adolescents who followed the brands in our sample (9.2%) was higher than the percentage of adolescents who followed any Twitter account (1.2%) (*p* < 0.001) (Table 3). We observed an even greater difference in the percentage of adolescents who followed the fast food brand accounts in our sample (12.7%) compared to the percentage of adolescents who followed any Twitter account (1.2%) (*p* < 0.001). 

We observed the opposite pattern for adults on Twitter. The percentage of adult Twitter users who followed the brands in our sample was either lower or not different compared to the percentage of adults who followed any Twitter account Table 3. For example, the percentage of users 45–54 years of age who followed the brands in our sample on Twitter (1.1%) was lower than the percentage of users 45–54 years of age who followed any account on Twitter (1.5%) *(p* = 0.004). 

But on Instagram, the brands in our sample had lower percentages of adolescent followers compared to other Instagram accounts. Specifically, the percentage of adolescents who followed the brands in our sample (7.0%) was lower than the percentage of adolescents who followed any Instagram account (11.5%) (*p* < 0.001). Similarly, the percentage of adolescents who followed the fast food brands in our sample (7.4%) was lower than the percentage of adolescents who followed any Instagram account (11.5%) (*p* = 0.01). For adult Instagram users, we observed the opposite pattern for all brands (e.g., the percentage of users 45–54 years of age who followed the food brands in our sample (1.1%) was higher than the percentage of users 45–54 years of age who followed any Instagram account (0.6%) (*p* < 0.001)).

### 3.2. Adolescent Followers of Sugary Drink Brands versus Low-Calorie Drink Brands

On Instagram, the percentage of adolescents who followed sugary drink brands (7.9%) was significantly higher than adolescents who followed low-calorie drink brands (4.3%) (*p* = 0.02) (Table 3). Similar percentages were also found on Twitter: we observed a higher percentage of adolescents who followed sugary drink brands (9.6%) compared to low-calorie drink brands (3.9%), but the difference was not statistically significant (*p* = 0.30).

We observed the opposite pattern for adults. On Instagram, the percentage of adults who followed sugary drink brands in our sample was either lower or not different than the percentage of adults who followed low-calorie drink brands. For example, the percentage of Instagram users 45–54 years of age who followed sugary drink brands (0.9%) was lower than the percentage of users 45–54 years of age who followed -calorie drink brands (1.7%) (*p* = 0.04).

### 3.3. Associations between Youth-Targeted Marketing Practices and the Percentage of Adolescent Followers

We found some significant differences in the percentages of adolescents who followed brands that heavily target youth compared to brands that do not heavily target youth. Specifically, total TV spending was positively associated with the percentage of adolescents who followed the brands in our sample on Twitter (β = 2.9; *p* = 0.03), but not on Instagram (β = 0.6; *p* = 0.21) Table 4. We did not, however, identify any associations between youth-targeted marketing ratios and the percentage of adolescent followers on social media. Specifically, the Child:Adult targeted ratio was not associated with the percentage of adolescents who followed our food and beverage brands on Twitter (*p* = 0.17) or Instagram (*p* = 0.24). The relationship was similar for the Adolescent:Adult targeted ratio.

## 4. Discussion

We purchased data from Demographics Pro to characterize the relationship between youth-targeted food marketing practices and the percentage of adolescents (age 13–17 years) who followed food/beverage brands as of January 2019. Our sample of 27 fast food, snack, and beverage brands collectively maintained 6.2 million adolescent followers on Twitter and Instagram. This figure may not represent unique social media users because it is likely that some adolescents followed multiple food/beverage accounts on both platforms, but the overall number of youth who followed these brands is concerning for several reasons. First, these adolescent followers are “opting in” to more exposure to food and beverage ads, which may increase their risk for poor dietary choices in response to ad exposure. Second, unhealthy brands had more adolescent followers than healthy brands. Monster Energy, for example, had the most adolescent followers (*n* = 3,198,430) on Twitter, followed by Wendy’s (*n* = 3,000,678), whereas the healthiest brands (e.g., Smart Water, Dasani Water, and Coca-Cola Life) had the fewest adolescent followers. Third, social media ads blur the line between entertainment and advertising, [11] which may increase adolescents’ susceptibility to this form of promotion.

Our results also demonstrate that the food and beverage brands in our sample had a higher percentage of adolescent followers compared to the percentage of adolescents who followed any account on Twitter. In addition, higher TV spending for a given brand was associated with a higher percentage of adolescents who followed that brand on Twitter, which provides support for the effectiveness of advertising in maintaining relationships with consumers. We did not, however, observe similar differences among adolescents using Instagram. The conflicting results on Instagram could be due to ceiling effects (i.e., 72% of adolescents in the US use Instagram [8]), which may translate to high numbers of adolescents who follow any type of account on Instagram. 

We also observed differences in the percentages of adolescents who followed sugary drink brands compared to low-calorie drink brands. Specifically, a higher percentage of adolescents followed sugary drink brands versus low-calorie drink brands on Instagram. Although this difference may be a result of companies’ history of spending more to promote sugary drinks compared to low-calorie drinks, [24] the high numbers of adolescents who follow sugary drink brands is a major public health concern. Given that sugary drink intake is associated with the development of type 2 diabetes and weight gain, [31,32] it is critical that public health policies address sugary drink promotion messages. 

This study contributes to food marketing research in several ways. Previous research has shown that adolescents view roughly 5700 food advertisements on television each year [4], and one study examining ad exposure among Canadian youth (ages 7–16 years) found that 72% of participants saw food and beverage ads on social media during a 10-minute data collection period [33]. The authors estimated that youth view almost 200 food/beverage ads per week on social media platforms. Our findings build upon those studies by demonstrating that 6.2 million adolescents are following food and beverage ads on Instagram and Twitter. Adolescents’ willingness to “opt in” for additional advertising exposure is concerning given that these brands post thousands of ads on social media annually [34]. The Federal Trade Commission should require disclosures (e.g., “This is advertising”) on posts made by food and beverage companies who manage these free social media accounts. Our findings also provide documentation that fast food, snack, and beverage brands with greater expenditures on advertising have a disproportionately higher percentage of youth followers compared to other accounts on Twitter [20,22,23,24]. Future studies should explore similar research questions using YouTube, which has a higher percentage of adolescent users relative to Instagram and Twitter [8]. Finally, we found that a higher percentage of females followed water brands compared to males, but the pattern was reversed for energy drinks. Demographics Pro has data on the intersection of followers who are female and age 13–17 years, but purchasing those data was outside the scope of our study. Still, the differences in followers among males and females is worthy of further attention given that the data suggests females may be more likely to follow low-calorie beverages, whereas males are more likely to follow sugary energy drink brands.

This study has some limitations and several strengths. Our methods are limited because of the need to use TV advertising as a proxy for which food and beverage brands target youth on social media. Although we were able to obtain Demographics Pro data on a majority of brands, we were not able to obtain data on all 27 brands across Instagram and Twitter. Further, the demographic data from Demographics Pro are inferences based on consumers’ networks, consumption, and language, which may not accurately reflect actual demographics of users. By including data from a company that uses proprietary algorithms, we are limited in our ability to achieve complete replicability of methods and detect errors. Data from Demographics Pro, however, is used by a wide range of well-known brands and companies, and their methods have been published in other academic research [27,35,36]. Additionally, capturing these data would be too time-intensive and cost-prohibitive for most researchers. Demographics Pro also filter out bots (i.e., artificial social media followers that brands can use to inflate their follower counts) by using proprietary clustering algorithms to identify small groups of bots that appear as followers among multiple target accounts, then link these small groups statistically to larger ‘bot farms’ (i.e., managed pools of fake followers). Another limitation is that we cannot be certain of the age of adolescents who use social media—most social media sites require a minimum age of 13 years to create an account, but it is possible for children to fabricate their age to meet this requirement. In addition, we cannot conclude whether youth-targeted marketing expenditures lead to more adolescent followers or whether adolescents’ interest in brands leads those brands to target youth more. Despite these limitations, our study is the first to document that 6.2 million adolescents follow one or more of the food and beverage brands from our sample. Our study also characterizes differences in the age of social media users who follow fast food, beverage, and snack brands on two popular social media platforms. Our methods are also strengthened by our inclusion of brands from those three brand categories, which spend more on youth-targeted advertising than other brands [20].

## 5. Conclusions

This study provides evidence that unhealthy food/beverage brands, especially fast food and sugary drink brands, maintain millions of adolescent followers on social media. The high percentage of adolescent followers is concerning because exposure to advertising is associated with a higher consumption of fast food, sugary drinks, and salty snacks among youth [37], which can increase the risk of obesity, heart disease, and other co-morbidities during adulthood [1]. With nearly ubiquitous use of social media among adolescents, youth are now exposed to food and beverage advertising across multiple digital domains. In fact, a recent narrative review of food marketing policies in 16 countries concluded that digital advertising needs to be included in policies aiming to reduce youth-targeted food marketing [38]. These data can also inform the proposed updates to the Children’s Online Privacy and Protection Act (COPPA), a federal policy in the United States that limits companies’ ability to collect online data from children younger than age 13 years and target children with targeted advertising [39]. In March 2019, two bipartisan US Senators proposed protecting adolescents up to age 15 years under COPPA. Given that 6.2 million adolescents ages 13–17 years followed the 27 brands in our sample, our data support the need to expand COPPA to include adolescents. 

## Figures and Tables

**Table 1:** Total numbers of followers of popular food and beverage brands (*n* = 27), by social media platform and user characteristics, 2019.

Brand	INSTAGRAM				TWITTER			
	Total Followers of Any Age (N)	Followers Who Have Used Platform >2 Years [N (%)]	Followers Who Post 1–7 Posts Per Week [N (%)]	Followers with Geo-Enabled Network Settings [N (%)]	Total Followers (N)	Followers Who Have Used Platform >2 Years [N (%)]	Followers Who Post 1–7 Posts Per Week [N (%)]	Followers with Geo-Enabled Network Settings [N (%)]
Burger King	1,623,786	596,979 (36.8)	667,387 (41.1)	459,521 (28.3)	1,713,262	984,029 (57.5)	569,187 (33.2)	533,582 (31.1)
Chick-fil-A	1,256,639	812,392 (64.6)	570,680 (45.4)	373,141 (29.7)	958,494	677,337 (70.7)	296,902	370,218 (38.6)
Coca Cola ^†^	2,592,532	1,119,247 (43.2)	1,078,960 (41.6)	745,042 (28.7)	-	-	-	-
Coca Cola Life	6049	2818 (46.6)	2675 (44.2)	1539 (25.4)	37,968	28,474	14,182 (37.4)	15,031 (39.6)
Coke Zero	98,742	29,992 (30.3)	38,760 (39.3)	13,886 (14.1)	253,914	238,469 (93.9)	86,676 (34.1)	967,52 (38.1)
Dairy Queen	473,337	241,847 (51.1)	206,677 (43.7)	87,300 (18.4)	477,430	426,219 (89.3)	152,802	179,847 (37.7)
Dasani Water ^‡^	-	-	-	-	14,327	12,588 (87.9)	4397 (30.7)	5260 (36.7)
Denny’s Diner ^‡^	-	-	-	-	520,034	396,021 (76.2)	158,581 (30.5)	205,970 (39.6)
Diet Coke	79,181	50,715 (64.1)	39,866 (50.3)	21,025 (26.6)	305,944	295,673 (96.7)	101,990 (33.3)	129,283 (42.3)
Dr. Pepper ^†^	536,521	234,595 (43.7)	221,669 (41.3)	104,732 (19.5)	-	-	-	-
Fanta	517,501	168,984 (32.6)	202,499 (39.1)	64,924 (12.5)	157,722	146,459 (92.9)	54,513 (34.6)	51,222 (32.5)
Gatorade	1,357,038	512,855 (43.9)	382,004 (32.7)	310,416 (26.6)	331,396	314,292 (94.9)	106,706 (32.2)	133,220 (40.2)
KFC ^†^	1,357,038	481,070 (35.4)	514,942 (37.9)	312,852 (23.1)	-	-	-	-
McDonald’s ^†^	3,342,259	1,366,449 (40.9)	1,324,871 (39.6)	846,327 (25.3)	-	-	-	-
Monster Energy	5,027,096	2,520,385 (50.1)	1,813,475 (36.1)	1,650,094 (32.8)	3,198,430	1,819,119 (56.9)	1,154,135 (36.1)	914,863 (28.6)
Mountain Dew	425,378	214,414 (50.5)	157,461	110,123 (25.9)	564,512	470,156 (83.2)	991,810 (33.1)	1,037,268 (34.6)
Oreo	2,506,211	1,070,202 (42.7)	1,080,879 (43.1)	685,348 (27.3)	834,516	567,998 (68.1)	268,371 (32.2)	289,157 (34.6)
Pepsi†	1,438,122	534,061 (37.2)	571,168 (39.7)	276,739 (19.2)	-	-	-	-
Pizza Hut ^†^	1,527,842	597,607 (39.1)	620,068 (40.6)	375,862 (24.6)	-	-	-	-
Red Bull	10,293,957	5,952,480 (57.8)	3,905,733 (37.9)	4,297,830 (41.8)	2,101,969	1,933,109	743,263 (35.4)	865,793 (41.2)
Smart Water	48,841	34,260 (70.1)	24,084 (49.3)	13,371 (27.4)	5493	4,992 (90.8)	1823 (33.2)	2453 (44.7)
Sprite	869,636	343,005 (39.5)	335,278 (38.6)	132,499 (15.2)	284,233	260,738 (91.8)	92,532 (32.6)	104,338 (36.7)
Starbucks ^†^	17,425,064	9,655,053 (55.4)	7,782,208 (44.7)	6,081,522 (34.9)	-	-	-	-
Subway	1,030,818	440,138 (42.7)	419,724 (40.7)	165,710 (16.1)	2,361,855	1,518,739 (64.3)	782,893 (33.1)	740,445 (31.4)
Taco Bell ^†^	1,274,017	758,805 (59.5)	537457 (42.2)	409673 (32.2)	-	-	-	-
Vitamin Water ^‡^	-	-	-	-	161,000	159,723 (99.2)	53,840 (33.4)	70,916
Wendy’s	834,654	400,339 (47.9)	357,171 (42.8)	147,687 (17.7)	3,000,678	2,088,191 (69.6)	991,810 (33.1)	1,037,268 (34.6)
Total	55,942,259	28,138,692	22,855,696	17,687,163	17,283,177	12,342,326	6,626,413	6,782,886

^†^ Instagram only. ^‡^ Twitter only.

**Table 2:** Total TV spending and targeted marketing characteristics of popular food and beverage brands (*n* = 27), 2019.

Brand	Followers Age 13–17 Years on Instagram [N (%)]	Followers Age 13–17 Years on Twitter [N (%)]	Female Followers on Instagram [N (%)]	Female Followers on Twitter [N (%)]	Total TV Spending ($000)	Child:Adult Targeted Ratio *	Adolescent:Adult Targeted Ratio *
Burger King	180,816 (11.1)	285,966 (16.7)	863,734 (53.2)	939,835 (54.9)	$298,148	0.40	0.52
Chick-fil-A	35,421 (2.8)	117,426 (12.3)	817,467 (65.1)	581,764 (60.7)	-	-	-
Coca Cola ^†^	225,492 (8.7)	-	1,467,833 (56.6)	-	$186,116	0.46	0.57
Coca Cola Life	194 (3.2)	2577 (6.8)	3420 (56.5)	22,305 (58.7)	$5,146	0.43	0.55
Coke Zero	9099 (9.2)	13,622 (5.4)	52,043 (52.7)	135,322 (53.3)	$53,752	0.39	0.50
Dairy Queen	20,243 (4.3)	32,533 (6.8)	305,406 (64.5)	300,732	$102,418	0.42	0.47
Dasani Water ^‡^	-	333 (2.3)	-	10,716 (74.8)	-	-	-
Denny’s Diner ^‡^	-	63,108 (12.1)	-	307,633 (59.2)	-	-	-
Diet Coke	1743 (2.2)	9989 (3.3)	56,074 (70.8)	211,145	$28,836	0.36	0.41
Dr. Pepper ^†^	39,971 (7.5)	-	263,723 (49.2)	-	-	-	-
Fanta	52,294 (10.1)	20,171 (12.8)	280,697 (54.2)	93,693 (59.4)	-	-	-
Gatorade	83,373 (7.1)	8472 (2.6)	274,075 (23.5)	110,100 (33.2)	$47,256	0.44	0.59
KFC ^†^	165,945 (12.2)	-	671,884 (49.5)	-	$193,500	0.41	0.51
McDonald’s ^†^	356,864 (10.7)	-	1,998,099 (59.8)	-	$597,346	1.16	0.76
Monster Energy	392,222 (7.8)	763,386 (23.9)	1,059,466 (21.1)	1,382,783 (43.2)	-	-	-
Mountain Dew	37,739 (8.9)	44,124 (7.8)	92,882 (21.8)	239,074 (42.4)	$45,214	0.40	0.56
Oreo	198,296 (7.9)	150,674 (18.1)	1,690,956 (67.5)	544,440 (65.2)	$26,222	0.36	0.40
Pepsi ^†^	119,843 (8.3)	-	726,715 (50.5)	-	$111,942	0.43	0.52
Pizza Hut ^†^	134,813 (8.8)	-	852,114 (55.8)	-	$177,801	0.48	0.57
Red Bull	507,065 (4.9)	78,553 (3.7)	2,287,180 (22.2)	692,749	-	-	-
Smart Water	1151 (2.4)	233 (4.2)	32,208 (65.9)	2917 (53.1)	-	-	-
Sprite	69,694 (8.0)	18,404 (6.5)	400,437	135,907 (47.8)	$20,484	0.46	0.65
Starbucks ^†^	973,448 (5.6)	-	13,400,081 (76.9)	-	-	-	-
Subway	70,857 (6.9)	319,289 (13.5)	594,909 (57.7)	1,344,122 (56.9)	$364,075	0.62	0.62
Taco Bell ^†^	-	2148 (1.3)	-	94,622 (58.8)	$362,077	0.40	0.55
Vitamin Water ^‡^	60,202 (4.7)	-	725,144 (56.9)	-	$6,180	0.56	0.80
Wendy’s	42,540 (5.1)	441,272 (14.7)	472,986 (56.7)	1,617,184 (53.9)	$234,938	0.41	0.52
Total	3,779,325	2,372,280	29,389,533	8,767,043	$2,861,451		

^†^ Instagram only. ^‡^ Twitter only.* Data Source: UConn Rudd Center. In [22], they calculated the ratios by dividing the ratio of TV ad exposure for children (or adolescents) versus adults by the ratio of TV viewing times.

**Table 3:** Comparison of the age of users who followed popular food and beverage brands (*n* = 27) and the age of the average Instagram and Twitter user, 2019.

	All Brands in Our Sample	*p*-Value ^†^	Fast Food Brands	*p*-Value ^†^	Drink Brands	*p*-Value ^†^	Sugary Drink Brands	*p*-Value ^†^	Low-Calorie Drink Brands	*p*-Value ^†^	Average on Platform
**Twitter (% followers)**											
*Age*											
Age 17 and under	9.2	**<0.001**	12.7	**<0.001**	6.7	**0.01**	9.6	**0.05**	3.9	**0.02**	1.2
Age 18 to 20	40.0	0.14	42.7	0.93	38.4	0.13	41.9	0.79	35.0	0.13	42.6
Age 21 to 24	25.8	**0.02**	23.4	**<0.001**	27.6	0.60	28.7	0.84	26.5	0.30	28.3
Age 25 to 29	13.2	**0.02**	11.4	**<0.001**	14.3	0.46	12.5	0.14	16.2	0.45	15.2
Age 30 to 34	6.5	0.81	5.2	**0.04**	7.1	0.79	4.3	**0.04**	10.0	0.26	6.7
Age 35 to 44	3.8	0.61	3.1	**0.02**	4.2	0.95	2.3 ^‡^	**0.02**	6 ^‡^	0.30	4.1
Age 45 to 54	1.1	**0.004**	1.1	**0.03**	1.1	0.07	0.6 ^‡^	0.11	1.6 ^‡^	0.64	1.5
Age 55 to 64	0.3	0.31	0.3	0.74	0.3	0.44	0.1 ^‡^	**<0.001**	0.4 ^‡^	0.29	0.3
Age 65 and over	0.2	0.42	0.1	**0.03**	0.2	0.89	0.1	**<0.002**	0.3	0.38	0.2
**Instagram** **(% followers)**											
*Age*											
Age 17 and under	7.0	**<0.001**	7.4	**0.01**	6.8	**<0.001**	7.9 ^‡^	**<0.001**	4.25 ^‡^	**0.02**	11.5
Age 18 to 20	30.6	0.07	34.1	0.46	27.8	**0.02**	31.3 ^‡^	0.26	28.3 ^‡^	0.17	33.1
Age 21 to 24	28.3	0.17	28.2	0.06	28.8	0.24	30.0	0.08	26.0	0.58	27.3
Age 25 to 29	17.8	0.80	16.6	0.09	18.8	0.26	17.5 ^‡^	0.69	21.5 ^‡^	0.08	17.9
Age 30 to 34	9.9	**0.001**	8.3	**0.01**	10.7	**0.01**	8 ^‡^	0.10	16.8 ^‡^	**0.01**	6.6
Age 35 to 44	5.1	**<0.001**	4.1	**0.02**	5.7	**0.01**	4 ^‡^	**0.05**	9.5 ^‡^	**0.04**	2.9
Age 45 to 54	1.1	**<0.001**	1.0	**0.02**	1.1	**0.02**	0.9 ^‡^	**0.05**	1.7 ^‡^	0.11	0.6
Age 55 to 64	0.2	**0.01**	0.2	**0.02**	0.2	0.09	0.1 ^‡^	0.30	0.4 ^‡^	0.16	0.1
Age 65 and over	0.2	**0.01**	0.2	**0.01**	0.1	0.08	0.1 ^‡^	0.34	0.2 ^‡^	0.14	0.1

^†^ The mean percentage of followers of the group of brands was statistically significantly different (*p* < 0.05) from the mean percentage of all Twitter/Instagram users using a one sample *t*-test. ^‡^ The mean percentage of followers of sugary drink brands was statistically significantly different (*p* < 0.05) compared to the mean percentage of followers of low-calorie drink brands using an independent samples *t*-test.

**Table 4:** Associations between targeted marketing and the percentage of users under 17 years of age and who followed popular food and beverage brands (*n* = 27) on Instagram and Twitter, 2019.

Type of Targeted Marketing Exposure	% Followers Age 17 and Under (Twitter)	*p*-Value	% Followers Age 17 and Under (Instagram)	*p*-Value
Total TV spending (in $10 million)	2.9	0.03 *	0.6	0.21
Child:Adult targeted ratio ^†^	−9.4	0.69	5.6	0.24
Adolescent:Adult targeted ratio ^†^	−20.8	0.20	11.4	0.17

^†^ The targeted ratios are calculated by dividing the ratio of TV ad exposure for children/adolescents vs. adults (or Black children/adolescents vs. White children/adolescents) by the ratio of TV viewing times. * This finding shows that greater spending on TV advertising per brand was associated with disproportionately higher percentage of followers under 17 years of age per brand.
